# Human antimicrobial peptide LL-37 is present in atherosclerotic plaques and induces death of vascular smooth muscle cells: a laboratory study

**DOI:** 10.1186/1471-2261-6-49

**Published:** 2006-12-20

**Authors:** Cristina D Ciornei, Hans Tapper, Anders Bjartell, Nils H Sternby, Mikael Bodelsson

**Affiliations:** 1 Department of Clinical Sciences, Division of Anaesthesiology and Intensive Care, Lund University, Lund, Sweden; 2 Department of Clinical Sciences, Division of Clinical and Experimental Infection Medicine (BMC), Lund University, Lund, Sweden; 3 Department of Clinical Sciences, Division of Urological Research, Lund University, Malmö University Hospital, Malmö, Sweden; 4 Department of Laboratory Medicine, Division of Pathology, Lund University, Malmö University Hospital, Malmö, Sweden

## Abstract

**Background:**

Death of smooth muscle cells in the atherosclerotic plaques makes the plaques more prone to rupture, which can initiate an acute ischemic event. The development of atherosclerosis includes the migration of immune cells e.g. monocytes/macrophages and T lymphocytes into the lesions. Immune cells can release antimicrobial peptides. One of these, human cathelicidin antimicrobial peptide hCAP-18, is cleaved by proteinase 3 generating a 4.5 kDa C-terminal fragment named LL-37, which has been shown to be cytotoxic. The aim of the study was to explore a potential role of LL-37 in the pathophysiology of atherosclerosis.

**Methods:**

We investigated the presence of LL-37 in human atherosclerotic lesions obtained at autopsy using immunohistochemistry. The direct effects of LL-37 on cultured vascular smooth muscle cells and isolated neutrophil granulocytes were investigated with morphological, biochemical and flow cytometry analysis.

**Results:**

The neointima of atherosclerotic plaques was found to contain LL-37-like immunoreactivity, mainly in macrophages. In cultured smooth muscle cells, LL-37 at 30 μg/ml caused cell shrinkage, membrane blebbing, nuclear condensation, DNA fragmentation and an increase in caspase-3 activity as studied by microscopy, ELISA and enzyme activity assay, respectively. Flow cytometry demonstrated that LL-37 in a subset of the cells caused a small but rapidly developing increase in membrane permeability to propidium iodide, followed by a gradual development of FITC-annexin V binding. Another cell population stained heavily with both propidium iodide and FITC-annexin V. Neutrophil granulocytes were resistant to these effects of LL-37.

**Conclusion:**

This study shows that LL-37 is present in atherosclerotic lesions and that it induces death of vascular smooth muscle cells. In a subset of cells, the changes indicate the development of apoptosis triggered by an initial mild perturbation of plasma membrane integrity. The findings suggest a role for LL-37 as a mediator of immune cell-induced death of vascular smooth muscle cells in atherosclerosis.

## Background

The smooth muscle cells are the principal source of collagen in the atherosclerotic plaque [1]. During the development of the plaques an increasing number of smooth muscle cells die, at least partly due to programmed cell death, apoptosis [2,3]. This impairs the collagen synthesis, which weakens the fibrous cap and makes the atherosclerotic plaques more prone to rupture [1]. The tissue exposed to the blood due to a plaque rupture is highly thrombogenic and can initiate an acute ischemic event [4].

An inflammatory response including the migration of immune cells, especially monocytes and T cells, into the lesion is pivotal to the development of atherosclerosis [5]. The weaponry of the cells of the innate immune system includes antimicrobial peptides, which can be divided into several groups based on their structure [6]. The cathelicidins comprise one group found in several mammalian species. They contain a highly conserved N-terminal domain, cathelin, and a variable C-terminal domain that constitutes the antimicrobial peptide [7]. The 18-kDa protein human cationic antimicrobial protein (hCAP-18) is the only human cathelicidin [8-10]. It is found in neutrophil granulocytes, lymphocytes and monocytes as well as in tissues e.g. squamous epithelium, the lung and the epididymis and is released extracellularly [11-15]. Liberated hCAP-18 is cleaved by proteinase 3, generating a 4.5 kDa C-terminal fragment named LL-37 [16]. Apart from having a broad antibacterial activity [17,18], LL-37 also exhibits lipopolysaccharide (LPS)-binding and LPS-neutralizing properties [17] as well as toxic effects on leukocytes and erythrocytes [19,20].

In an earlier study, aiming to investigate the effects of LL-37 on nitric oxide synthesis in the blood vessel wall, we found that LL-37 non-specifically inhibits the expression of inducible nitric oxide synthase in vascular smooth muscle cells. The inhibition is accompanied by DNA fragmentation, suggesting that it is caused by cell death, presumably apoptosis [21].

Since apoptosis of vascular smooth muscle cells has been implicated as an important factor in the pathophysiology of atheromatous disease [22], we investigated the presence of LL-37 in atherosclerotic plaques as well as morphological and biochemical effects of LL-37 on cultured vascular smooth muscle cells. The results indicate that LL-37 is present in the neointima of atherosclerotic lesions and that it induces death of smooth muscle cells at concentrations lower than those previously reported to be cytotoxic.

## Methods

### Immunohistochemistry

Paraffin-embedded archival material of human aorta obtained from autopsy within 24 hours after death of four persons at the Department of Pathology, Malmö University Hospital was subjected to immunohistochemistry. Tissues were routine-fixed in 4% buffered paraformaldehyde and embedded in paraffin. Sections, 3.5 μm in thickness, were prepared. Haematoxylin-eosin stained slides were examined by a National Board-certified pathologist (NHS) and atherosclerotic lesions were identified on the basis of intimal thickening and the presence of foam cells. Immunostaining for hCAP-18/LL-37 was performed using DAKO ChemMate™ detection kit (code K5001) and a DAKO TechMate™ 500/1000 staining machine (BioTek solutions, Winooski, VT, USA). Briefly, the sections were deparaffinized and rehydrated. For antigen retrieval, tissue sections were incubated with citrate buffer (10 mM, pH 6.0) and heated in a microwave oven at 750 W for 2 × 5 min. The sections were incubated with a Protein A-purified polyclonal immunoglobulin G (Ig G) fraction raised against recombinant hCAP-18 [23] at a final dilution of 0.5 μg/mL followed by biotinylated mouse anti-rabbit IgGs. The antibodies against hCAP-18 recognize both intact hCAP-18 and the active C-terminal fragment LL-37 [15]. Immunoreactivity was visualized using the manufacturer's protocol with horseradish peroxidase (SA-HRP) and 3,3'diaminobenzidine tetrahydrochloride (DAB) as chromophore. Then, sections were counterstained with Mayer's hematoxylin solution and coverslips applied with Faramount™ mounting medium (DAKO A/S). As a negative control, adjacent sections were processed by replacing the primary antibody with non-immune rabbit IgG (DAKO) used at same concentration as the primary anti-hCAP-18 polyclonal IgGs used. Double staining of separate sections of the lesions was performed using the antibody raised against hCAP-18 and the ChemMate™ EnVision™ Detektion Kit (DAKO, code K5007) yielding a brown colour and a monoclonal mouse anti-human CD68 antibody (DAKO, Code M 0814) and ChemMate™ Detektion kit, APAAP (DAKO, code K5000) yielding a red colour.

### Isolation and culture of vascular smooth muscle cells

The Review Board for the care of animal subjects approved the study (The Animal Research Ethics Council of Malmö/Lund, Approval No. M145-03). Vascular smooth muscle cells were isolated from the thoracic aorta of a male Sprague Dawley rat by the explant method [24,25]. The cells were identified as smooth muscle cells by their characteristic hill and valley appearance in culture and by their expression of an approximately 40-kDa protein with immunoreactivity corresponding to smooth muscle α-actin as determined by Western blot using a monoclonal anti-smooth muscle α-actin antibody (A-2547, Sigma-Aldrich St. Louis, MO, USA). Primary human aortic smooth muscle cells (CC-2571) were obtained from Bio Whittaker, Walkersville, Md. At confluence, the cells were harvested using 0.025 % trypsin and 0.01 % v/v ethylenediaminetetraacetic acid (EDTA, both from Sigma-Aldrich), rinsed in Hanks' Balanced Salt Solution, seeded at a density of 25 % and further cultured in Dulbecco's Modified Eagle's Medium (DMEM) containing fetal bovine serum (10 %), penicillin (100 U/ml), streptomycin (100 μg/ml) and amphotericin-B (250 ng/ml, all from Life Technologies, Täby, Sweden). The subsequent experiments were performed with cells from passage 3–6 or 5–7 for rat and human cells, respectively.

### Isolation of human neutrophils

Human neutrophils were isolated from heparinized blood from healthy donors using the Polymorph isolation kit (Nycomed Pharma AS Diagnostics, Oslo, Norway). Following the manufacturer's instructions, whole blood was layered carefully on neutrophil isolation medium and centrifuged at 400 × g for 30 min at room temperature. After centrifugation the following fractions were apparent: mononuclear cells, neutrophils and erythrocyte pellet. The polymorphonuclear leukocytes layer was suspended in PBS, followed by 10 min centrifugation at 350 × g; residual erythrocytes were removed by hypotonic lysis. After two washes in PBS with centrifugation for 5 min at 250 × g, the pellet was suspended in Minimum essential medium (MEM) and neutrophils were counted using a hemocytometer. The concentration of cells was adjusted to 10^7 ^cells/ml.

### Microscopy of cultured cells

Rat aorta smooth muscle cells were grown on methanol-cleansed coverslips in 6-well plates and incubated for 2 or 5 h in serum free DMEM with/without LL-37 (10 or 30 μg/ml) or camptothecin (5 μg/ml, Sigma-Aldrich). After culture, the coverslips were washed with ice-cold PBS containing 1 mM Ca^2+ ^and the cells were then stained at room temperature for 20 min with FITC-annexin V (20 μg/ml, Roche Molecular Biochemicals, Mannheim, Germany) and 4'6-diamino-2-phenyliondole dihydrochloride (DAPI) nucleic acid stain (100 nM, Molecular probes, Eugene, OR, USA). Thereafter, the cells were fixed with 1 % paraformaldehyde (Becton Dickinson, Bedford MA, U.S.A) in ice-cold PBS containing 1 mM Ca^2+^. Fixation was initiated on ice for 15 min and continued at room temperature for 45 min. Cover slips were then mounted using ProLong AntiFade Reagent (Molecular Probes). Visual inspection and recording of images was performed using a Nikon Eclipse TE300 inverted fluorescence microscope equipped with a Hamamatsu C4742-95 cooled CCD camera, using a Plan Apochromat 60 × objective.

### Measurement of caspase-3 activity

Confluent rat aorta smooth muscle cells cultured on 24-well plates were incubated for 1, 3 or 7 h in serum free DMEM in the absence (control) or presence of LL-37 (10 or 30 μg/ml). The caspase-3 activity was measured by using a fluorometric immunosorbent enzyme assay (FIENA, Roche Molecular Biochemicals) according to the manufacturer's instructions. Briefly, the caspase-3 from cellular lysates was captured by immobilized monoclonal anti-caspase-3 antibodies followed by addition of a substrate that is cleaved by caspase-3 generating a fluorescent compound. Fluorescence was then measured at 400 nm excitation and 505 nm emission. Simultaneous measurement of lactate dehydrogenase (LDH) activity in the medium (see below) indicated that a fraction of the cells lysed during the incubation. The caspase-3 activity values were therefore corrected in order to correspond to the fraction of intact cells using the formula

VIC=VC1−VLVLT
 MathType@MTEF@5@5@+=feaafiart1ev1aaatCvAUfKttLearuWrP9MDH5MBPbIqV92AaeXatLxBI9gBaebbnrfifHhDYfgasaacH8akY=wiFfYdH8Gipec8Eeeu0xXdbba9frFj0=OqFfea0dXdd9vqai=hGuQ8kuc9pgc9s8qqaq=dirpe0xb9q8qiLsFr0=vr0=vr0dc8meaabaqaciaacaGaaeqabaqabeGadaaakeaacqWGwbGvdaWgaaWcbaacbiGae8xsaKKae83qameabeaakiabg2da9maalaaabaGae8Nvay1aaSbaaSqaaiab=neadbqabaaakeaaieaacqGFXaqmcqGHsisldaWcbaWcbaGae8Nvay1aaSbaaWqaaiab=XeambqabaaaleaacqWFwbGvdaWgaaadbaGae8htaWKae8hvaqfabeaaaaaaaaaa@3BF2@

Where *V*_*IC *_is the caspase-3 activity of intact cells, *V*_*C *_is the caspase-3 activity of the sample, *V*_*L *_is the LDH activity of the sample and *V*_*LT *_is the total LDH activity of lysed cells. The caspase-3 activity is expressed as % of untreated controls.

### Measurement of DNA fragmentation

Confluent rat or human aorta smooth muscle cells cultured on 24-well plates were incubated in serum free DMEM for 16 h in the absence (control) or presence of LL-37 (10 and 30 μg/ml). Internucleosomal DNA fragmentation was measured using a Cell Death Detection ELISA Kit (Roche Molecular Biochemicals) according to the manufacturer's instructions. In short, the cells were lysed in the culture wells and the DNA fragments in the lysate were bound to a microtiter plate coated with monoclonal anti-histone antibodies. The bound DNA fragments were then detected by peroxidase-conjugated monoclonal anti-DNA antibodies and 2,2'-azino-di- [3-ethylbenzthiazoline sulfonate]. Optical density was measured at 415 nm and is expressed as % of untreated controls.

### LDH assay

Confluent rat aorta smooth muscle cells cultured on 24-well plates were incubated for 1, 3, or 7 h in serum free DMEM in the absence (control) or presence of LL-37 (10 or 30 μg/ml). The lactate dehydrogenase (LDH) activity released into the medium was measured by using the *in vitro *toxicology assay kit (Tox-7, Sigma-Aldrich) according to the manufacturer's instructions. In short, the medium was centrifuged at 2000 × g for 4 min. The LDH in the supernatants converted a tetrazolium dye, which was measured spectrophotometrically at 490 nm. The LDH activity is expressed as % of total LDH activity of lysed cells.

### Flow cytometry

Rat aorta smooth muscle cells were harvested from culture plates using trypsin/EDTA and washed in PBS once followed by a 2-h incubation in bicarbonate free MEM at 37°C using gentle end-over-end rotation. Thereafter, LL-37 (30 μg/ml) was added and cells were incubated for 10, 30, 60, or 120 min followed by pelleting and re-suspension in cold calcium-containing PBS. Staining was performed for 20 min on ice with FITC-annexin V (30 μg/ml) and propidium iodide (30 μg/ml, BD Pharmingen, San Diego, CA, USA) in calcium-containing PBS. Fluorescence-activated cell sorter (FACS) analysis was performed immediately (Becton Dickinson FACS Calibur instrument). 10,000 cells/sample were counted, and results were evaluated by using the Cell Quest software (Becton Dickinson). The same protocol was used for staining of human neutrophils incubated with LL-37 (30 μg/ml) for 30 or 120 min.

### Statistical analysis

DNA fragmentation was analyzed using Kruskal-Wallis one way ANOVA on ranks followed by Dunnett's post hoc test. LDH and caspase-3 activity was analyzed using one way repeated measures ANOVA followed by Holm-Sidak test. Significance was accepted at *P *< 0.05. Values are means ± S.E.M.; 'n' equals number of independent experiments.

## Results

### LL-37 is present in atherosclerotic plaques

**Figure 1:**
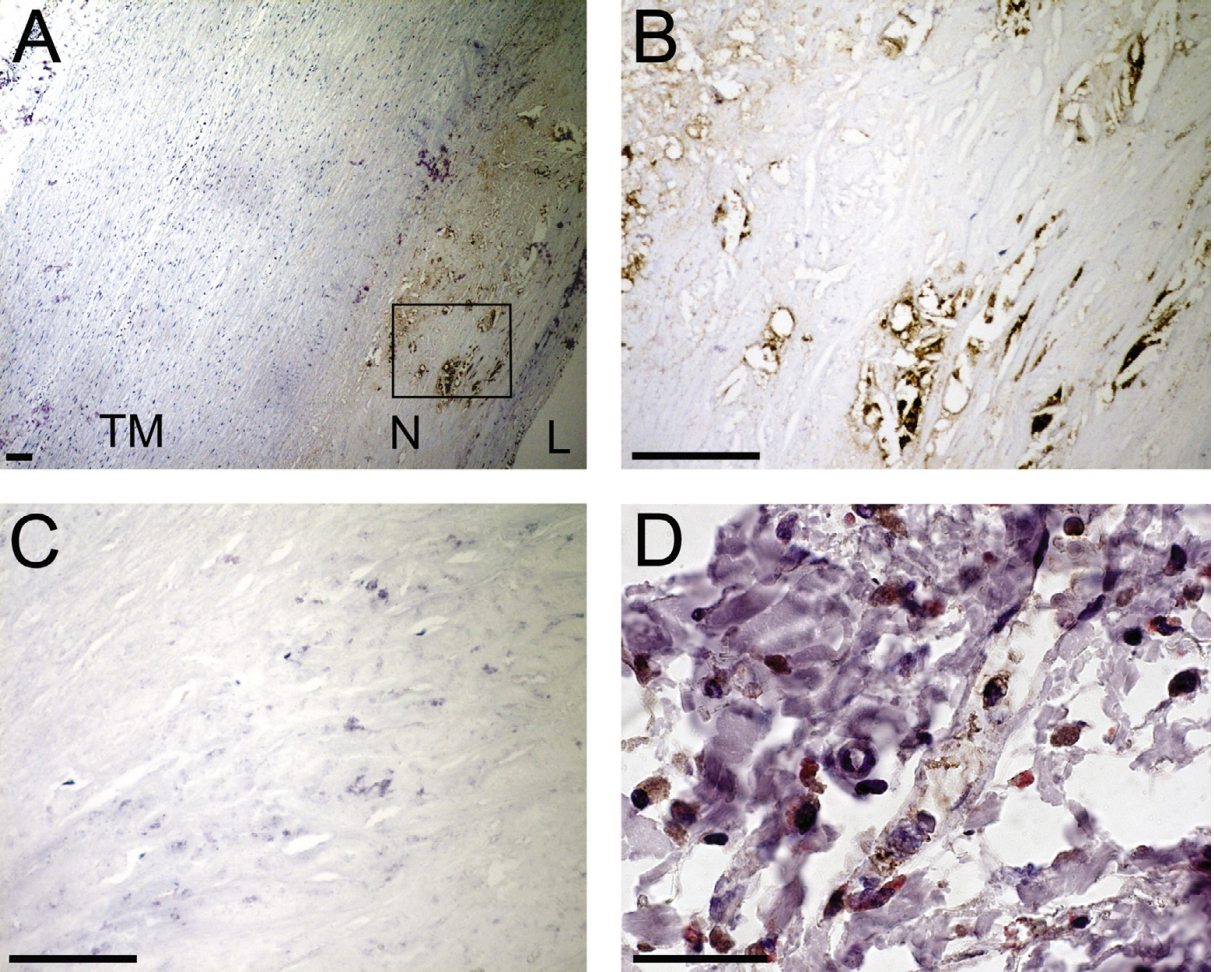
**Detection of hCAP-18/LL-37 by immunohistochemistry in sections of human aorta**. (A) Low power view. Binding of the specific antibody was detected by peroxidase, which yields a brown color reaction against a pale background. Dense binding was found in the neointima (N) of an athreosclerotic lesion. The vascular lumen (L) and the tunica muscularis (TM) are indicated. (bar, 100 μm). (B) Portion of the neointima indicated in (A). hCAP-18/LL-37-like immunoreactivity is found (bar, 100 μm). (C) Control, where the specific antibody has been replaced by nonimmune serum, resulting in loss of labeling (bar, 100 μm). (D) High power image of a slide double stained for hCAP-18/LL-37 (brown) and the macrophage marker CB68 (red). HCAP-18/LL-37 and CD68 immunoreactivity are mainly co-localized to the same cells (bar, 10 μm).

Immunohistochemistry demonstrated dense labeling in atherosclerotic lesions of all specimens (fig. 1). The staining was most intense in the neointima, i.e. hyperplastic portions of the intima laying adjacent to the vascular lumen. In portions of the aortic wall with a normal appearance no labeling was observed. Double staining showed that most of the hCAP-18/LL-37 labeling was present in CD68 positive cells, i.e. in macrophages.

### LL-37 induces morphological changes and disrupts the cell membrane phospholipid asymmetry

**Figure 2:**
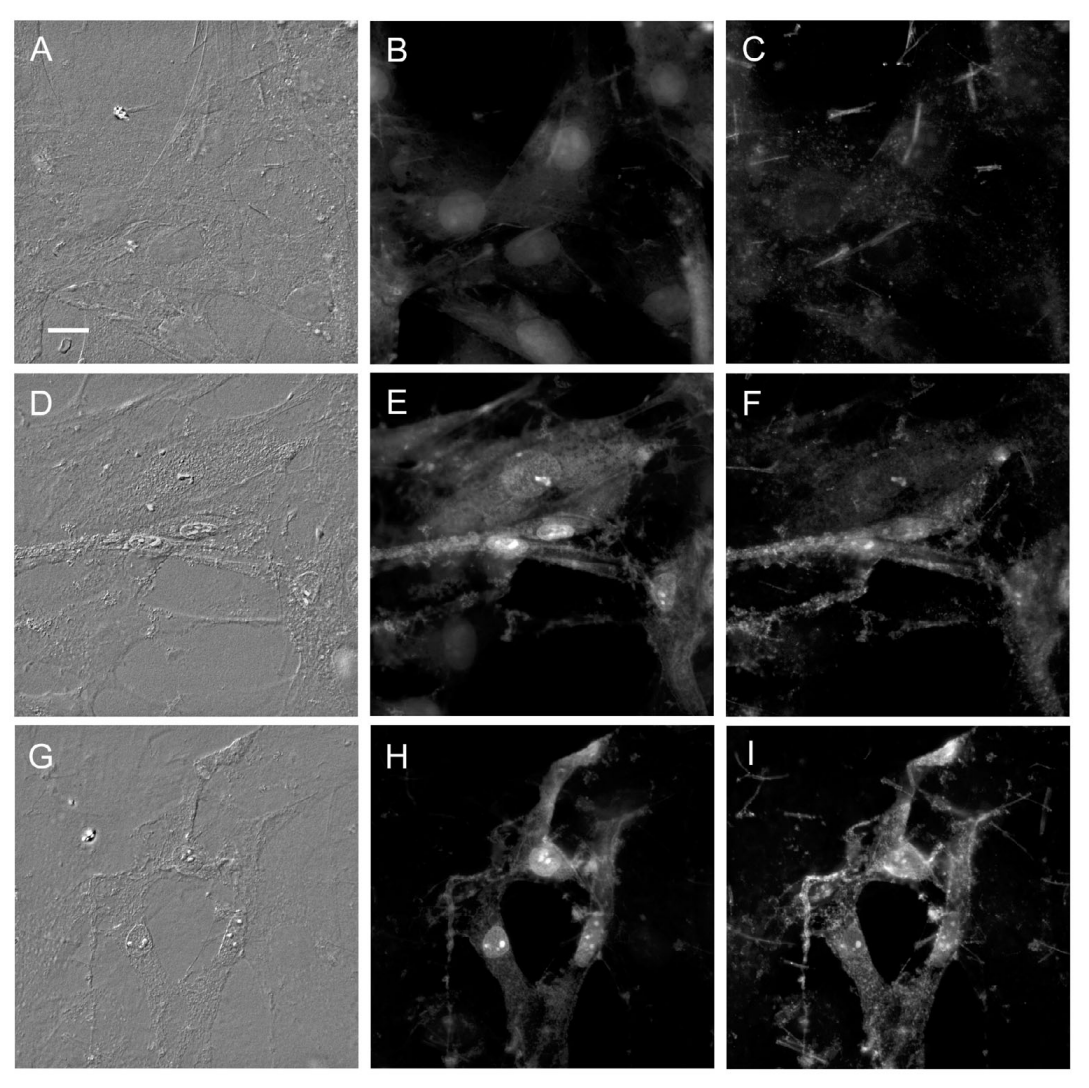
**Microscopic images of cultured vascular smooth muscle cells**. Cells were incubated in the absence (control, A, B, C) or presence of LL-37 (30 μg/ml) for 2 (D, E, F) or 5 (G, H, I) hours. The images show cells viewed with differential interface contrast (Nomarski) microscopy (A, D, G), stained with 4'6-diamino-2-phenyliondole dihydrochloride (DAPI), (B, E, H), or FITC-annexin V (C, F, I). Control cells have a smooth surface (A) with large evenly stained nuclei (B) and no FITC-annexin V staining of the cell membrane (C). After 2 hours' incubation with LL-37, some cells appear shrunken with a rough surface (D), smaller nuclei with fragmented chromatin (E), and a cell membrane staining with FITC-annexin V (F) indicating apoptosis. After 5 h the apoptotic changes are more marked (G, H, I; bar, 10 μm).

Exposure of cultured smooth muscle cells to LL-37 at 10 μg/ml caused only small and inconsistent morphological changes (not shown). Exposure to LL-37 at 30 μg/ml for 2 or 5 h resulted in progressive cell shrinkage and a rough appearance of the cell membrane (fig. 2).

DAPI is a membrane-permeable fluorochrome that stains DNA/RNA. As demonstrated in fig. 2, the nuclei of most cells appeared smaller with fragmented chromatin after 2 or 5 h incubation with LL-37 (30 μg/ml).

A reduction in cell volume, nuclear condensation and chromatin fragmentation are typical changes seen during cell death via the apoptosis pathway [26,27] and has previously been observed in vascular smooth muscle cells during apoptosis induced by cytokines [28], or by over-expression of tissue inhibitor of metalloproteinase-3 [29].

During early stages of apoptosis in vascular smooth muscle cells, phosphatidylserine is exposed on the outer surface of the cell membrane where it can then be stained with FITC-annexin V [30-32]. The plasma membrane of most cells incubated with LL-37 (30 μg/ml) for 2 or 5 h bound FITC-annexin V (fig. 2). No staining was seen in control cells.

Taken together, the results of the morphological studies suggest that LL-37 induces cell death via an apoptosis-like mechanism. The cellular changes induced by LL-37 (30 μg/ml) were similar to those induced by camptothecin, a compound well known to induce apoptosis (not shown) [33].

### LL-37 increases the caspase-3 activity

**Figure 3:**
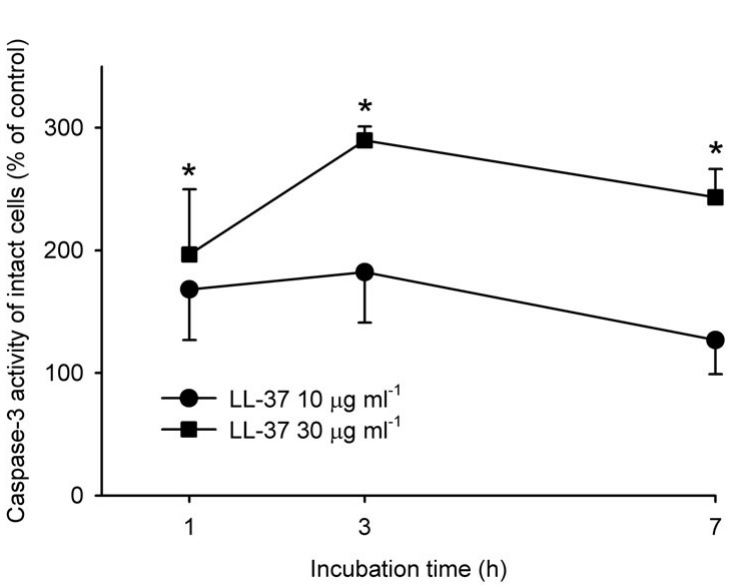
**Caspase-3 activity in cultured vascular smooth muscle cells**. Cells were incubated for 1, 3 or 7 hours in the presence of LL-37 at 10 (●) or 30 (■) μg/ml. A significant increase in caspase-3 activity was observed in cells incubated for 1, 3 and 7 hours with LL-37 at 30 μg/ml (*n *= 6, *P < 0.05, one way repeated measures ANOVA followed by Holm-Sidak test). Values are means + or - S.E.M.

Although apoptosis was originally characterized on the basis of changes in cell morphology, these are secondary to biochemical alterations [34]. One feature of apoptosis is an increase in caspase-3 activity, also demonstrated in smooth muscle cells [32]. LL-37 (30 μg/ml) induced an increase in caspase-3 activity after 1 h incubation, which persisted for 7 h (*P *< 0.05). This supports the morphological results suggesting that vascular smooth muscle cells exposed to LL-37 undergo apoptosis (fig. 3).

### LL-37 induces DNA fragmentation

**Figure 4:**
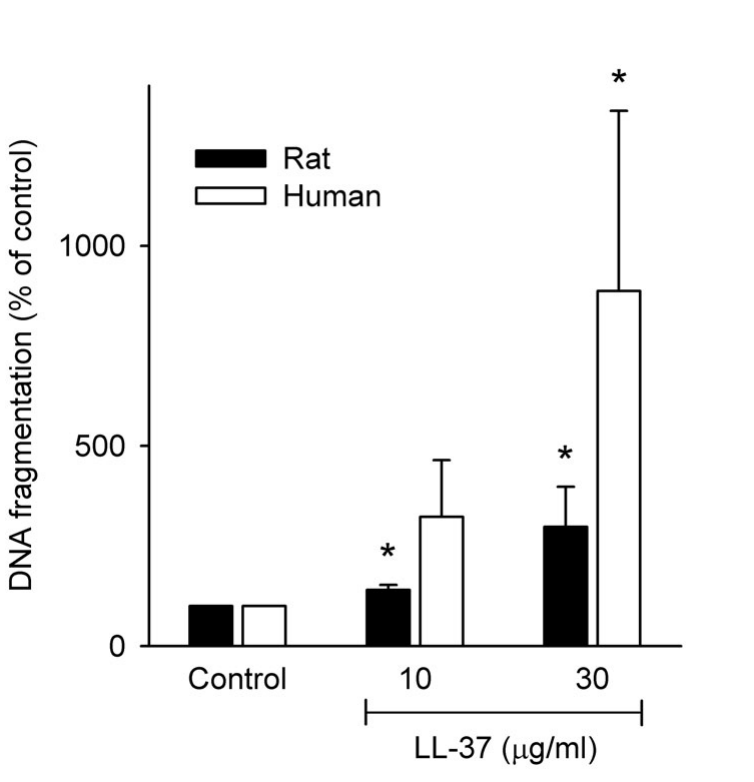
**Effect of LL-37 on DNA fragmentation in cultured rat and human aortic smooth muscle cells**. Rat (filled bars) and human (open bars) cells were analyzed after 16 h incubation. LL-37 at 10 and 30 μg/ml induced a statistically significant DNA fragmentation in rat cells compared to controls and LL-37 at 30 μg/ml induced a statistically significant DNA fragmentation in human cells compared to controls (*n *= 7, * *P *< 0.05, Kruskal-Wallis one way ANOVA on ranks followed by Dunnett's test). Values are means + S.E.M.

Incubation with LL-37 for 16 h caused a significant internucleosomal DNA fragmentation in both rat and human cultured aortic smooth muscle cells (*P *< 0.05, fig. 4). The degree of fragmentation induced by 30 μg/ml was similar to that induced by cytokines in cultured human vascular smooth muscle cells [28]. A statistically significant fragmentation compared to control was found at an LL-37 concentration as low as 10 μg/ml. This shows that LL-37 can induce apoptosis-like cell death at low concentrations, similar to the MIC value for several bacterial strains [17-19].

### LL-37 causes cellular leakage of LDH activity

**Figure 5:**
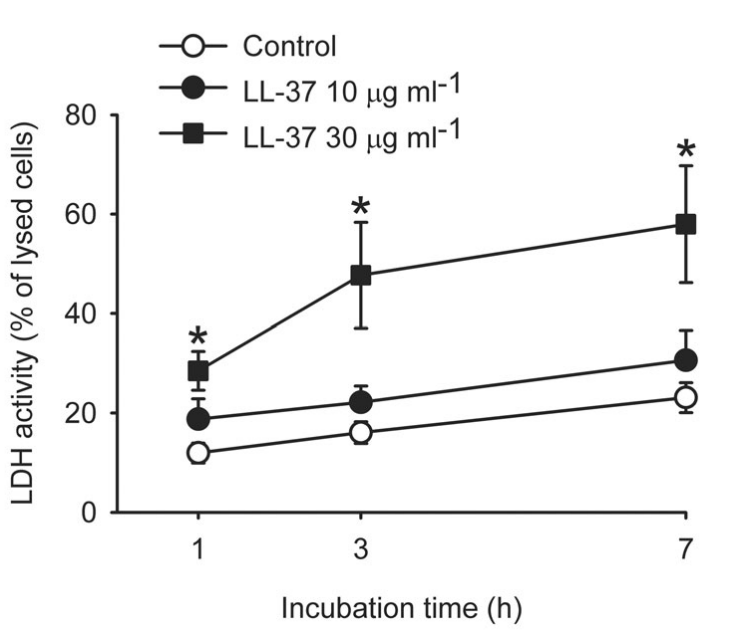
**Release of LDH from cultured vascular smooth muscle cells**. Cells were incubated for 1, 3 or 7 hours without (Control, ○) or with LL-37 at 10 (●) or 30 (■) μg/ml. The release of LDH was significantly increased from cells incubated for 1, 3 and 7 hours with LL-37 at 30 μg/ml (*n *= 7, *P < 0.05, One way repeated measures ANOVA followed by Holm-Sidak test). Values are means ± S.E.M.

Fig. 5 shows that the release of LDH activity from the vascular smooth muscle cells into the cell culture medium was significantly higher from cells incubated with LL-37 (30 μg/ml), as compared to controls (*P *< 0.05). The LDH increase, observed already after incubation with LL-37 for one h, suggests a loss of cell membrane integrity.

### Flow cytometry

**Figure 6:**
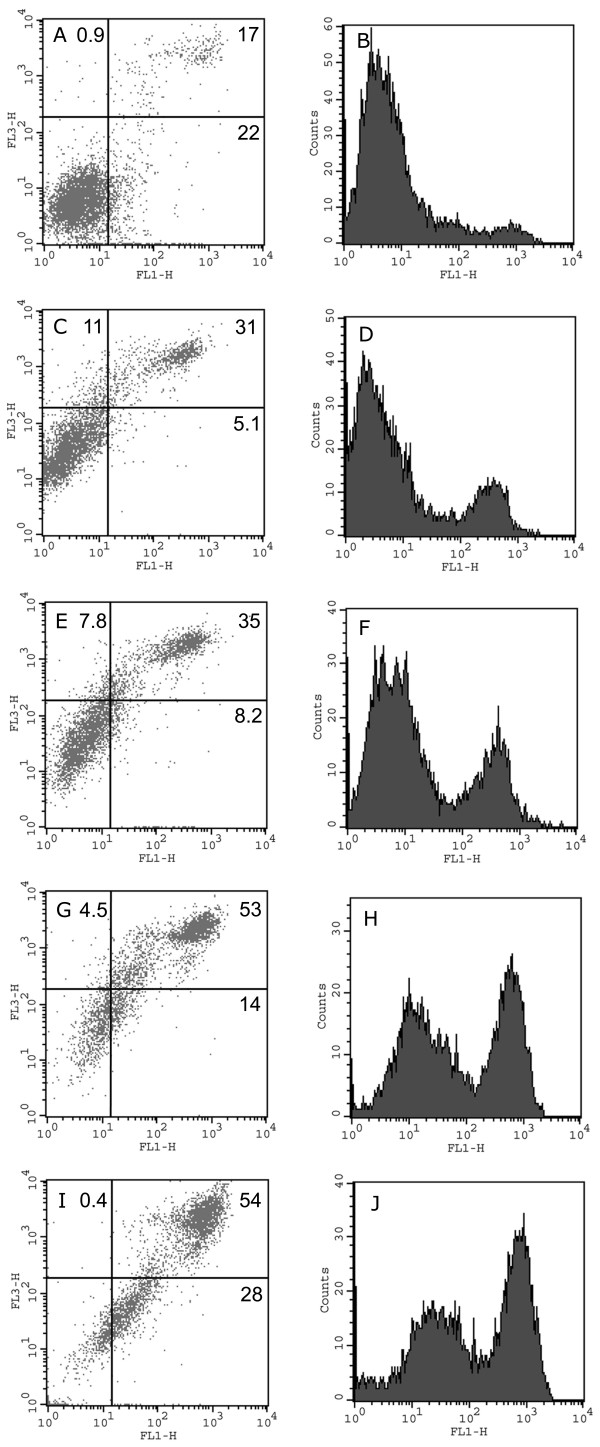
**Flow cytometry of cultured vascular smooth muscle cells**. Dot-plots (left) of FITC-annexin V (x-axis)/propidium iodide (y-axis) fluorescence and corresponding histograms for FITC-annexin V fluorescence (right) of vascular smooth muscle cells incubated in the absence (A, B, control) or presence of LL-37 (30 μg/ml) for 10 min (C, D), 30 min (E, F), 60 min (G, H) or 180 min (I, J). The diagrams on the left are divided into four areas and the percentages of the total number of stained cells within each area are given. Viable control cells are found in the lower left part if the diagrams and non-viable cells, heavily stained with FITC-annexin V and propidium iodide, are found in the upper right part of the diagrams. Apoptotic cells, stained with FITC-annexin V but not propidium iodide, are found in the lower right part of the diagrams. Incubation with LL-37 resulted, within 10 min, in a moderate increase in propidium iodide staining (C) of the viable cells followed by a gradually developing increase in FITC annexin V staining from 5.1 % at 10 minutes to 28 % at 180 minutes indicating the development of apoptosis (E-J). The fraction of non-viable cells increased from 17 to 54 % during the whole time course studied.

Finally, we followed up these results by flow cytometry analysis of suspended vascular smooth muscle cells (fig. 6). Cells with an intact plasma membrane are impermeable to propidium iodide while extensive membrane damage, such as due to necrosis, markedly increases the influx and staining of intracellular components by propidium iodide. Also, FITC-annexin V enters necrotic cells, staining internal membranes by binding to phosphatidylserine. However, during apoptosis, phosphatidylserine is exposed on the outer surface of the cell membrane and FITC-annexin V thus also stains apoptotic cells. Thus, dual color flow cytometry with FITC-annexin V/propidium iodide can be used to assess the cell fractions undergoing apoptosis and necrosis, respectively.

In a diagram plotting the FITC-annexin V signal on the x-axis and the propidium iodide signal on the y-axis, normal cells, stained weakly with both FITC-annexin V and propidium iodide, can be found in the lower left part of the diagram. Necrotic cells stain heavily with both FITC-annexin V and propidium iodide and will therefore be plotted in the upper right part of the diagram. Finally, apoptotic cells display mainly a FITC-annexin V fluorescence and therefore resides in the lower right part of the diagram. In the present study, untreated control cells generally displayed low staining for both FITC-annexin V and propidium iodide except for a small population of necrotic cells (17%, fig. 6A). This pattern was not changed during 180 min incubation (not shown). Incubation with LL-37 (30 μg/ml) resulted within 10 minutes in an increase in propidium iodide staining of the viable cells, indicating a rapidly developing LL-37-induced permeability increase (fig. 6C). The increase was only 10-fold, which is a moderate increase compared to the increase in the propidium iodide fluorescence signal of the necrotic cells, which was about 1000-fold. The propidium iodide staining of this population then remained constant during the following 170 min. The FITC-annexin V staining of the moderately propidium iodide-positive cell population, still residing in the lower left part of the diagram at 10 minutes (fig. 6C), increased gradually during 180 minutes, indicating the development of apoptosis (fig. 6E, G and 6I).

This rightward shift transferred these cells into the lower right part of the diagram, the percentage of cells in this part increasing from 5.1 % at 10 minutes to 28 % at 180 minutes. The gradual rightward shift of these cells is illustrated as the leftmost peak in the histograms of FITC-annexin V fluorescence (fig. 6D, F, H and 6J). This peak was shifted from about 2 fluorescence units at 10 minutes to 30 units at 180 minutes. This represents a 15 fold increase in FITC-annexin V fluorescence. The magnitude of this rightward shift was similar to the shift observed in vascular smooth muscle cells undergoing apoptosis in response to serum deprivation [31] or generation of reactive oxygen species [32]. A growing cell population that stained heavily with both propidium iodide and FITC-annexin V can, however, be observed in the upper right part of the diagrams fig. 6A, C, E, G and 6I, indicating that the number of severely damaged cells increased in parallel to the development of apoptosis. This cell population may explain the early rise in LDH activity and the changes could be either secondary to the apoptotic events or represent a subset of cells undergoing severe membrane damage due to immediate cytolysis. In contrast, human neutrophils incubated with LL-37 (30 μg/ml) showed weak staining with propidium iodide and FITC-annexin V after both 30 and 120 minutes' incubation time, indicating that neutrophils are resistant to LL-37 at this concentration (not shown).

## Discussion

The present results show that the human cathelicidin antimicrobial peptide LL-37 is present in atherosclerotic plaques of human aorta and that it induces changes in cultured vascular smooth muscle cells some of which are typical for apoptosis. Apoptosis, also termed programmed cell death, is essential in normal tissue development and homeostasis [33], but it has also an important role in the pathophysiology of common conditions such as atherosclerosis. Recently Lau and colleagues demonstrated that LL-37 at similar concentrations induces apoptosis in human lung and airway epithelial cells [35].

Apart from its bactericidal action, LL-37 has previously been found to be cytotoxic to eukaryotic cells such as human peripheral leukocytes and the T-cell line MOLT [19] and also to cause lysis of human red blood cells [20,36]. However, the concentrations required were higher than those inducing the apoptosis-like changes in vascular smooth muscle cells and airway epithelial cells, suggesting a different mechanism of action. It cannot be determined on the basis of the present results whether the concentrations required to induce the changes are pathophysiologically relevant. We found a statistically significant DNA fragmentation induced by LL-37 at 10 μg/ml, while a statistically significant increase in caspase-3 activity as well as distinct morphological cellular changes within the time frame investigated required 30 μg/ml. Sørensen and colleagues [23] found a plasma level of LL-37 around 1.2 μg/ml, which is considerably lower than the levels required in the present study. However, the mean levels of LL-37 have been shown to double in tracheal aspirates of newborn infants during infection [37]. This indicates that levels of LL-37 are locally increased during inflammation and activation of the immune system. It is supported by the finding of a large increase in LL-37 expression in human skin after sterile incision [38]. Furthermore, infection of mice skin with group A *streptococci *increases the local expression of the corresponding murine cathelicidin CRAMP [38]. These results suggest that the local levels of LL-37, at a site of inflammation, such in an atheroma, may reach the range of those demonstrated to be harmful to the smooth muscle cells used in the present study.

Disruption of the plasma membrane integrity can trigger apoptosis [33]. Cathelicidins, including LL-37, have been shown to bind to the negatively charged bacterial membrane by means of their positive charge [39]. However, LL-37 also binds to the zwitterionic membranes of eukaryotic cells, probably due to its hydrophobicity [18,20,40]. The present results suggest that this binding results in an increase in membrane permeability as LL-37 induced a small but rapidly developing increase in propidium iodide staining, already before extensive membrane damage was observed. However, the flow cytometry results showed that one population of cells gradually progressed to a state with more severe membrane damage, allowing efficient staining of both DNA and internal membranes. Such deteriorated cells, could detach from the surface and in the analysis of adherent cells, this population might be underestimated.

Risso and colleagues have shown that the bovine cathelicidins BMAP-27 and BMAP-28 induce DNA fragmentation and morphological alterations in human activated lymphocytes and U937 cells, indicating apoptosis [41]. They also found that apoptosis is preceded by an increase in membrane permeability to propidium iodide. Thus, human and bovine cathelicidins may induce apoptosis via a similar mechanism. It has further been shown that human antimicrobial peptides of the well characterized class α-defensins induce a rapidly developing membrane permeabilization in K562 cells, followed by a second phase, possibly representing apoptosis [42]. Both α-defensins and the frog antimicrobial peptides magainins form channels in eukaryotic cell membranes [43,44]. It has been demonstrated that LL-37 forms oligomers in eukaryotic membranes [20], but it remains to be determined whether these represent channels.

In the present study, neutrophil granulocytes were not affected by LL-37 even after prolonged exposure at the same concentration that seemed to induce apotosis in vascular smooth muscle cells. In fact, the results from two recent studies suggest that LL-37 can inhibit spontaneous neutrophil granulocyte apoptosis [45,46]. This protective effect of LL-37 seems to be mediated, at least partly, by the chemoattractant receptor formyl receptor-like peptide 1 (FPRL1) [46]. One reason for the different action of LL-37 on neutrophils and vascular smooth muscle cells could be that vascular smooth muscle cells do not express FPRL1 (unpublished observations). This hypothesis is supported by the observation that LL-37 does not induce apoptosis but proliferation in FPRL1-expressing human umbilical vein endothelial cells [47].

Onset and progression of atherosclerosis requires the recruitment of immune cells into the atherogenic foci [4,5]. Monocytes migrate into the atherosclerotic lesions and are to a large extent retained there in their mature form, macrophages [48]. In the present study we demonstrated the presence of hCAP-18/LL-37-like immunoreactivity in atherosclerotic plaques. These results confirm those of a recently published study by Edfeldt and colleagues who demonstrated the expression of LL-37 in artherosclerotic material obtained from patients undergoing carotid endarterectomy [49]. As in the present study, LL-37 immunoreactivity was mainly found in macrophages, which have previously been found to synthesize and release LL-37 [11]. Serial staining demonstrated that LL-37 was also expressed in some specimens in endothelial but not in smooth muscle cells [49]. Edfeldt and colleagues found that *Chlamydia peumoniae*, which has been associated with atherosclerotic disease [50], is resistant to the antimicrobial action of LL-37, possibly one reason for its survival in the atherosclerotic lesion [49].

Vascular smooth muscle cells co-cultured with monocytes undergo apoptosis via direct cell/cell contact resulting in Fas-ligand/Fas interactions [51]. However, this monocyte-induced apoptosis is promoted by soluble macrophage-derived pro-apoptotic factors such as nitric oxide [52] and tumor necrosis factor-α [53]. The present results suggest that LL-37 could be another substance contributing to the monocyte/macrophage-induced apoptosis of smooth muscle cells. Smooth muscle cell apoptosis is predominant in the region underlying a plaque rupture [2,54]. The involvement of LL-37 in this process is based on the assumption that LL-37 is released in such advanced lesions. The specimens examined in the present study as well as those demonstrated by Edfeldt and colleagues [49] all represent lesions compatible with an earlier stage of the disease, fibroatheromas. Thus, any differential expression of LL-37 during the development of an atheroma as well as in more advanced lesions needs to be subject of further study. However, macrophages have been demonstrated to accumulate adjacent to thinning or rupture of the fibrous cap of advanced lesions [55]. The spatial association between LL-37 containing cells and smooth muscle cell apoptosis also needs to be addressed.

Apart from being implicated in plaque rupture, smooth muscle cell apoptosis has been found to precede the calcification of atherosclerotic lesions [56]. Furthermore, the immune cells are recruited to the atherosclerotic lesions via expression of cell adhesion molecules and the chemotactic activity of oxidized LDL and IL-8 [4]. Agerberth and colleagues and Yang and colleagues have demonstrated that LL-37 has a chemotactic function in T-cells and monocytes via activation of the formyl peptide receptor-like 1 (FPRL1) [11,57]. It seems plausible that the release of LL-37 could contribute to attracting more of these cells into the atherosclerotic plaques.

The present findings of the morphological changes, the time-dependent-binding of FITC-annexin V, the increase in caspase-3 activity and the DNA fragmentation all point to that LL-37 induces apoptosis in a subset of cultured vascular smooth muscle cells. However, the parallel leak of LDH from the cells and the flow cytometry results suggest that there could be another cell population that die from necrosis. Why some cells apparently undergo apoptosis, while other cells die from necrosis cannot be determined on the basis of the present results, but the faith of a cell may be cell cycle dependent. This is supported by the finding that LL-37 analogs induce apoptosis in an oral squamous carcinoma cell line but not in normal gingival fibroblasts or human keratinocytes [58].

## Conclusion

We have demonstrated that LL-37 is present in atherosclerotic lesions and induces death of cultured vascular smooth muscle cells, mainly via an effect on the plasma membrane permeability leading to apoptosis. These findings suggest a role for LL-37 as a mediator of immune cell-induced apoptosis of vascular smooth muscle cells in atherosclerosis.

## Competing interests

The author(s) declare that they have no competing interests.

## Authors' contributions

CDC participated in the design and coordination of the study, carried out the isolation, culture and biochemical studies on smooth muscle cells and helped to draft the manuscript. HT carried out the microscopy and flow cytometry of cultured cells and interpreted the data obtained. AB: carried out and interpreted the immunohistochemistry. NHS: carried out and interpreted the microscopy of paraffin sections. MB: conceived of the study, and participated in its design and coordination and drafted the manuscript. All authors read and approved the final manuscript.

## Pre-publication history

The pre-publication history for this paper can be accessed here:


